# Measured active rotational-vibrational energy levels (MARVEL) analysis of high-resolution rovibrational spectra of H^12^C^14^N

**DOI:** 10.1038/s42004-026-02031-5

**Published:** 2026-04-28

**Authors:** Waed O. H. Al-Nashash, Ala’a A. A. Azzam, Sana A. E. Abzakh, Dunia Alatoom, Mohammad Taha I. Ibrahim, Jonathan Tennyson, Tibor Furtenbacher, Attila G. Császár

**Affiliations:** 1 Research Department, AstroJo Institute, Amman, Jordan; 2 Department of Physics, The University of Jordan, Amman, Jordan; 3 Department of Physics and Astronomy, University College London, London, UK; 4 DevConsult Zrt., Budapest, Hungary; 5 ELTE Eötvös Loránd University, Institute of Chemistry, Budapest, Hungary; 6 Present Address: Department of Physics, Khalifa University, Abu Dhabi, United Arab Emirates

**Keywords:** Physical chemistry, Computational chemistry

## Abstract

Studying the rovibrational spectra of hydrogen cyanide (HCN) has become increasingly relevant due to growing concerns about the molecule’s environmental and health risks and its special role in astronomy. Empirical rovibrational energy levels are determined based on the MARVEL (Measured Active Rotational-Vibrational Energy Levels) protocol for H^12^C^14^N, the most abundant HCN isotopologue. The spectroscopic analysis is based on 23 225 measured transitions, of which 14 728 are unique, collected from 39 literature sources. In contrast to most previous MARVEL studies, which utilized a very large number of experimental sources, for H^12^C^14^N 70 % of the measured transitions are contained in two literature sources. The experimental transitions used in the final MARVEL analysis form a spectroscopic network with a single principal and just a few floating components, but a large number of orphans. To ensure the reliability of the final empirical rovibrational energy level set, the final MARVEL analysis involved artificial transitions determined from accurately fitted effective Hamiltonian models. The transitions collected and validated span the spectral range of 0 − 13 018 cm^−1^. Altogether 5564 empirical rovibrational energies, which make ca. 30% of states determined through effective Hamiltonian fits for the [H,C,N] system, with an average expanded uncertainty of 0.006 cm^−1^, are obtained for H^12^C^14^N; they are associated with 174 vibrational bands. A comparison of the empirical rovibrational energy levels obtained in this study, and the transitions that can be generated from them, with those in the composite experimental/empirical/first-principles-computed HITRAN2020, ExoMol, and MOMeNT-90 datasets reveals excellent overall agreement. Nevertheless, there are particularly notable exceptions in the case of HITRAN2020, for which there appear to be problems with the published labels of many lines.

## Abstract (web summary)

Studying the rovibrational spectra of HCN has become increasingly relevant due to growing concerns about the molecule’s environmental and health risks. Here, the authors use the MARVEL protocol to study the most abundant isotopologue of HCN, H^12^C^14^N, reporting 5,564 accurate empirical rovibrational energy levels with well-defined expanded (two-sigma) uncertainties obtained from 22,647 validated transitions.

## Introduction

The electronic ground state of the triatomic [H, C, N] molecular system is famous for having two stable, readily observable isomers, HCN and HNC, both with a linear equilibrium structure belonging to the *C*_*∞v*_ point group. Compared to the lower-energy isomer, hydrogen cyanide, H–C ≡ N, HNC has a zero-point-corrected relative energy of (*hc*)5112 cm^−1^ ^1^ (see also the discussion of Makhnev et al.^2^), a value consistent with an earlier estimate of (*hc*)5126 ± 50 cm^−1^ ^3^, computed within the framework of the focal-point analysis^4,5^ scheme. The two [H, C, N] isomers are separated by a barrier of (*hc*)16 733 ± 100 cm^−1^ ^3^.

High-resolution spectroscopic investigation of the [H, C, N] system is well within the reach of modern broadband spectrometers. The isomerizing [H, C, N] system, along with its two constituent molecules HCN and HNC, has attracted the attention of a significant number of molecular physicists and quantum chemists^2,3,6–18^, spectroscopists^11,19–79^, and astronomers^80–97^. Astronomers expressed interest, not least because the branching ratio between HCN and HNC, observed in outer space, may vary from 80:1 to 1:5^16,98^.

Hydrogen cyanide was one of the first molecules discovered in interstellar space during the early stages of radio astronomy, and it remains one of the most common molecules observed in the interstellar medium^80^. HCN has been detected, for example, in remote galaxies^81^, interstellar clouds^88^, particularly in star-forming regions^89^, planetary nebulae^93^, interplanetary dust^85^, comets^86^, and in the atmospheres of outer planets and their moons, such as Titan, Saturn’s largest moon^87,90^. HCN is also the most abundant molecule after H_2_ and CO in carbon-rich stars^99^. HCN has also been well studied in the atmospheres of exoplanets^91,94,95^. The importance of studying the rovibrational spectra of HCN is partly due to growing concerns about the molecule’s environmental and health risks. Satellites, such as NASA’s Aura (with the Tropospheric Emission Spectrometer, TES) and the Infrared Atmospheric Sounding Interferometer (IASI), use HCN spectra to track wildfire plumes^100^. Spectroscopic laboratory studies are somewhat hindered by the fact that HCN is a highly toxic substance and that it is classified as a chemical warfare agent^101^. Nevertheless, in 2011, as a pinnacle of previous studies, Mellau^74^ reported a complete set of “experimental” relative rovibrational energies for H^12^C^14^N up to (*hc*)6880 cm^−1^.

The primary objective of the present study has been to provide two comprehensive spectroscopic datasets for the hydrogen cyanide isotopologue H^12^C^14^N (occasionally denoted in the rest of this study as 124). The first dataset delivered contains a collection of accurately measured line positions, each with unique lower- and upper-state labels, taken from the literature and validated in the present study. The experimental transitions for H^12^C^14^N were collected from a large number of sources^22–34,36–53,55,57–71,74,78^. The second dataset contains accurate empirical rovibrational energies with well-defined expanded (two-sigma) uncertainties obtained from the first dataset. The empirical energy levels have been determined from the measured and validated rovibrational transitions using the MARVEL (Measured Active Rotational-Vibrational Energy Levels) procedure^102–107^, which relies on the theory of spectroscopic networks^108,109^. This paper is the first to report results of our ongoing investigation of the spectroscopy of hydrogen cyanide isotopologues and continues our larger-scale project aimed at calculating empirical rovibrational energy levels for the major isotopologues of various molecules of astronomical and atmospheric interest; for example, for the nine most abundant isotopologues of water^110–116^, 12 isotopologues of carbon dioxide^117–124^, and six isotopologues of carbon monoxide^125,126^; these datasets are forming the input for machine learning activities aimed at improving the prediction of transition wavenumbers for the minor isotopologues^127^. The data produced for H^12^C^14^N is expected to help (a) refine theoretical and computational spectroscopic models, and (b) improve line-by-line spectroscopic databases, including ExoMol^18,128^, HITRAN2020^129^, and MOMeNT-90^79^.

## Results and discussion

### Energy levels

As part of the Supplementary Information for this paper, the empirical rovibrational energy values obtained in this study are available in the file “EnergyLevels_124.txt”. Each energy level in this data file is characterized by (a) a rovibrational label, (b) an empirical (MARVEL) energy level (in *hc* cm^−1^), (c) an expanded (two-sigma) energy uncertainty (in *hc* cm^−1^), and (d) the number of transitions in which this energy level participates.

**Fig. 1:**
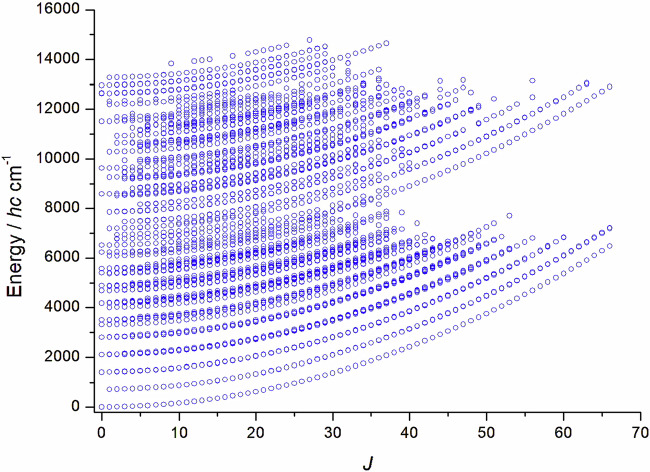
Empirical energies of the rovibrational quantum states of the H^12^C^14^N molecule, determined by the MARVEL procedure, as a function of the rotational quantum number *J*.

Empirical energies of the rovibrational quantum states of H^12^C^14^N, determined in this study as a function of the end-over-end rotational quantum number *J*, are shown in Fig. 1. Each dotted, quasi-quadratic curve in Fig. 1 corresponds to a distinct vibrational band origin. The MARVEL analysis yielded rovibrational energy levels with *J* values of up to 66 for the vibrational ground state.

**Fig. 2:**
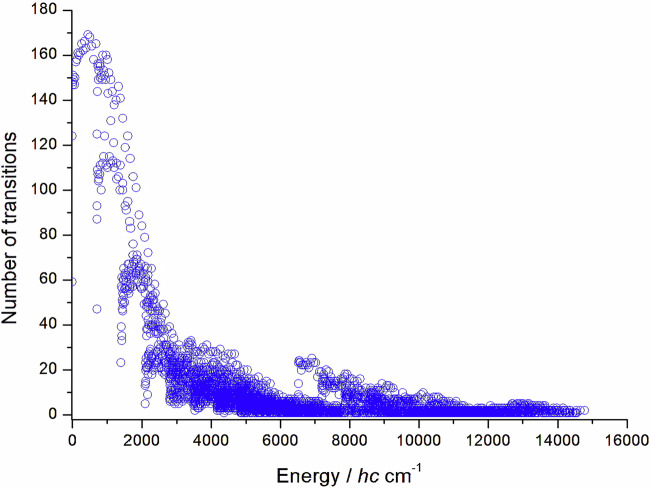
Number of measured transitions incident to each quantum state of H^12^C^14^N *versus* the empirical rovibrational energies determined in this study.

Figure 2 shows the degree distribution of the spectroscopic network of H^12^C^14^N, which represents the number of transitions incident on each quantum state. The distribution follows the expected behavior^105,130^, namely, it is heavy-tailed and power-law-like. The first five largest hubs, that is, the states with the largest number of incident transitions^108^, are in the ground vibrational state with *J* = 17, 18, 15, 13, and 16.

**Fig. 3:**
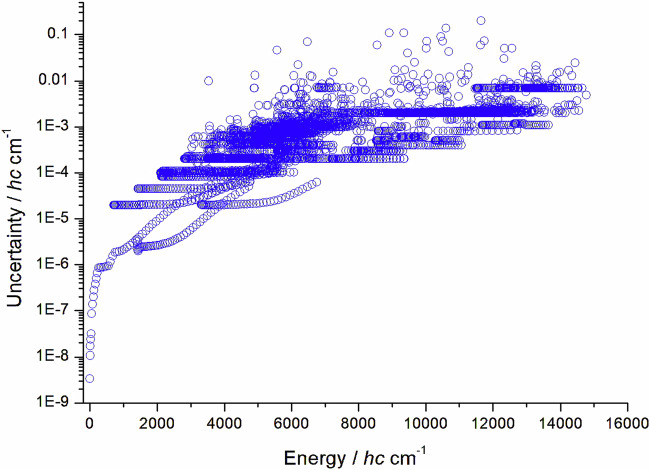
Distribution of the expanded uncertainties of the empirical rovibrational energy levels of H^12^C^14^N determined in this study against the energy values.

Figure 3 shows the expanded (two-sigma) uncertainties of the empirical rovibrational energy levels determined in this study for H^12^C^14^N against the energy level values determined through the final MARVEL analysis. It can be seen that the uncertainties of the ground state energy levels, up to about (*hc*)1000 cm^−1^, are less than (*hc*) 1 × 10^−6^ cm^−1^, and that the average uncertainty calculated for the total energy set is (*hc*) 3 × 10^−3^ cm^−1^.

**Fig. 4:**
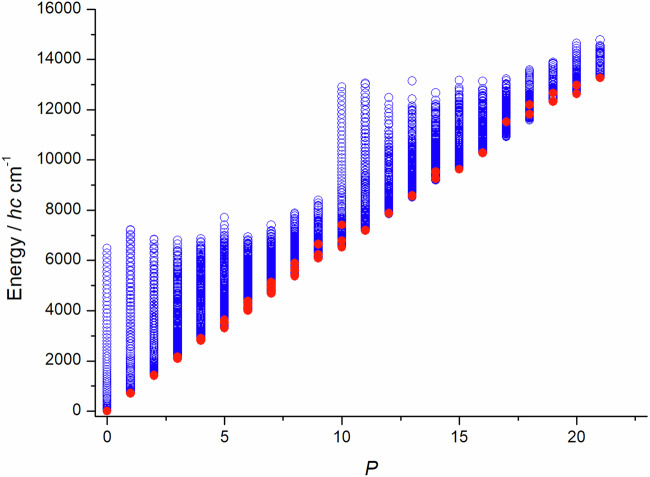
Empirical rovibrational energy levels of H^12^C^14^N determined in this study, grouped according to the polyad number *P* = 5*v*_1_ + *v*_2_ + 3*v*_3_. The lowest-energy quantum states within each vibrational band, which are called vibrational band origins even in cases when *J* ≠ 0 for them, are indicated as red dots.

Figure 4 displays the distribution of the empirical rovibrational energy values as a function of the *P* = 5*v*_1_ + *v*_2_ + 3*v*_3_ polyad values, with a maximum value of ${P}_{\max }=21$. As expected, this plot shows significant overlaps among the rovibrational energies corresponding to different *P* values; nevertheless, the polyad scheme works very well where this is expected, that is, for the lowest-energy states of each vibrational band.

### Comparison with other datasets

Let us start by providing a brief history of the rovibrational spectroscopic data that will be compared with the results of the present study. The original line list provided in 2002 for the [H, C, N] system by the UCL group^131^ contained only less accurate first-principles results^3,132^. This line list was subsequently improved in several steps. In 2006, Harris et al.^17^ replaced a relatively small number of first-principles energy levels with their more accurate empirical counterparts. Then, in 2014, a much more systematic replacement of the first-principles energy levels by experimental/empirical ones was performed^18^, using accurate energies from effective Hamiltonian fits conducted by Mellau^74–76^. This line list, which is provided as part of the ExoMol project^128^, is still referred to as ‘Harris’, as 06HaTeKaPa^17^ is the source of the original first-principles transition data. All these line lists provide transitions for both the HCN and HNC isomers and are based on a large number of first-principles energy levels, the accuracy of which is far from that of the experimental/empirical levels.

In 2021, under the leadership of Mellau, an HCN line list called MOMeNT-90, covering the limited spectral region 0 − 7500 cm^−1^, was published^79^. The line positions of this list were calculated using only experimentally known energy levels of H^12^C^14^N. The latest edition of the HITRAN database, HITRAN2020^129^, contains a H^12^C^14^N line list based on the MOMeNT-90 dataset.

#### Vibrational band origins

The lowest-energy quantum states of each rovibrational progression of H^12^C^14^N may differ from the typically defined vibrational band origins (VBO), for which *J* = 0, since when *l*_2_ ≠ 0, *J* cannot be smaller than *l*_2_. Therefore, in this study, VBOs are defined in a more general sense: not as quantum states with *J* = 0, but as the lowest-energy quantum states for each rovibrational progression. Tables 1–3 list the VBOs of H^12^C^14^N for the cases *J* = *l*_2_ = 0, *J* = *l*_2_ = 1, and *J* = *l*_2_ = 2, respectively. The three tables together define all the VBOs of H^12^C^14^N with *l*_2_ < 3 up to 10,000 cm^−1^, including 25, 35, and 28 VBOs determined during the present investigation for *J* = 0, 1, and 2, respectively.

**Table 1:** Vibrational band origins of H^12^C^14^N with *J* = *l*_2_ = 0 and energies up to (*hc*)10,000 cm^−1^; the expanded (2*σ*) uncertainties in the last digits are given in parentheses

(*v*_1_ *v*_2_ *l*_2_ *v*_3_)	06HaTeKaPa ^17^^*a*^	MARVEL this work	11Mellau ^74^	21MeMaGoZo ^79^	ExoMol database^*b*^
(0 0 0 0)	0.000	0.000 000(0)	0.000 000(0)^*c*^	0.000 00	0.000 00
(0 2 0 0)	1414.9	1411.413 374(4)	1411.413 450(60)^*c*,*e*^	1411.413 45	1411.413 45
(0 0 0 1)	2100.6	2096.845 6(1)	2096.845 547(56)^*c*,*f*^	2096.845 54	2096.845 54
(0 4 0 0)	2801.5	2802.958 9(1)	2802.958 744(86)^*c*,*g*^	2802.958 74	2802.958 74
(1 0 0 0)	3307.7	3311.476 82(2)	3311.477 09(18)^*c*,*h*^	3311.477 08	3311.477 08
(0 2 0 1)	3511.0	3502.119 7(2)	3502.121 10(17)^*c*,*i*^	3502.121 10	3502.121 10
(0 0 0 2)	4176.2	4173.070 8(2)	4173.070 91(37)^*c*,*j*^	4173.070 91	4173.070 91
(0 6 0 0)	4181.5	4174.609 5(5)	4174.608 61(20)^*c*^	4174.608 60	4174.608 60
(1 2 0 0)	4686.3	4684.310 1(2)	4684.309 98(12)^*c*,*k*^	4684.309 97	4684.309 97
(0 4 0 1)	4891.8	4888.028(7)	4888.039 81(55)^*c*^	4888.039 28	4888.039 28
(1 0 0 1)	5394.4	5393.696(1)	5393.697 73(13)^*c*^	5393.697 73	5393.697 73
(0 8 0 0)	5537.8	−	5525.812 84(57)^*c*^	5525.812 84	5525.812 84
(0 2 0 2)	5586.5	5571.733 6(3)	5571.734 31(61)^*c*,*l*^	5571.734 30	5571.734 30
(1 4 0 0)	6033.7	−	6036.960 11(40)^*c*^	6036.960 11	6036.960 11
(0 0 0 3)	6242.4	6228.598 4(4)	6228.598 30(12)^*c*,*m*^	6228.598 29	6228.598 29
(0 6 0 1)	6260.6	−	6254.405 902(2118)^*c*^	6254.405 90	6254.405 90
(2 0 0 0)	6513.5	6519.610 4(2)	6519.610 486(94)^*c*,*n*^	−	6519.610 45
(1 2 0 1)	6768.5	−	6760.705 136(264)^*c*^	6760.705 13	6760.705 13
(0 10 0 0)	6879.6	−	6855.443 088(1064)^*c*^	6855.443 08	6855.443 08
(0 4 0 2)	6961.0	−	−	6951.683 03	6951.683 03
(1 6 0 0)	7369.2	−	7369.443 75(36)^*d*^	7369.443 75	7369.443 75
(1 0 0 2)	7461.6	−	−	7455.423 74	7455.423 47
(0 8 0 1)	7617.2	−	−	−	7600.535 81
(0 2 0 3)	7641.3	−	−	−	*7641.282 59*
(2 2 0 0)	7855.8	−	−	−	7853.510 85
(1 4 0 1)	8110.3	−	8107.969 38(108)^*d*^	8107.969 38	8107.969 38
(0 12 0 0)	8197.5	−	−	−	8161.892 00
(0 0 0 4)	8283.4	−	−	−	*8283.369 86*
(0 6 0 2)	8323.5	−	−	−	*8323.518 65*
(2 0 0 1)	8584.6	8585.581 2(4)	− ^*o*^	−	8585.581 35
(1 8 0 0)	8691.7	−	8681.196 53(184)^*d*^	8681.196 53	8681.196 53
(1 2 0 2)	8830.3	−	−	−	*8830.275 95*
(0 10 0 1)	8954.5	−	−	−	*8954.463 46*
(0 4 0 3)	9009.0	−	−	−	*9008.999 67*
(2 4 0 0)	9164.1	−	−	−	9167.088 13
(1 6 0 1)	9440.1	−	9435.374 50(800)^*d*^	9435.374 50	9435.374 50
(0 14 0 0)	9488.5	−	−	−	*9488.538 60*
(1 0 0 3)	9508.9	−	−	−	*9508.925 14*
(3 0 0 0)	9619.2	9627.086 9(4)	− ^*p*^	−	9627.087 40
(0 2 0 4)	9674.7	−	−	−	*9674.669 69*
(0 8 0 2)	9675.3	−	−	−	*9675.253 29*
(2 2 0 1)	9922.9	−	−	−	9914.399 17
(1 10 0 0)	9994.0	−	−	−	*9993.952 74*

^*a*^ Downloaded from https://cdsarc.cds.unistra.fr/ftp/VI/121/.

^*b*^ The italicized entries of this column correspond to first-principles computed values.

^*c*^ The data are taken from Tables I and II of ref. ^74^.

^*d*^ The data are taken from Table 4 of ref. ^76^.

^*e*^ Band center measured in absorption at 1411.413 375(26)^61^.

^*f*^ Band center measured in absorption at 2096.845 558(27)^61^.

^*g*^ Band center measured in absorption at 2802.958 821(38)^61^.

^*h*^ Band center measured in absorption at 3311.476 829(49)^61^.

^*i*^ Band center measured in absorption at 3502.119 692(61)^61^.

^*j*^ Band centers measured in absorption at 4173.070 839(77)^61^.

^*k*^ Band center measured in absorption at 4684.310 004(60)^61^.

^*l*^ Band center measured in absorption at 5571.733 70(12)^61^.

^*m*^ Band center measured in absorption at 6228.598 30(12)^61^.

^*n*^ Band center measured in absorption at 6519.610 318(60)^61^.

^*o*^ Band center measured in absorption at 8585.581 07(12)^61^.

^*p*^ Band center measured in absorption at 9627.086 85(22)^61^.

There are a number of lessons that can be learned from the three sets of VBOs. First, the first-principles computed 06HaTeKaPa^17^ VBOs deviate significantly from the accurate, empirical VBOs of the present study; for slightly higher vibrational excitations, the errors can be in the 10 − 20 cm^−1^ range, suggesting that the accuracy of the underlying PES is relatively low by today’s standards. Second, there are only a few cases where the VBOs determined in this study deviate outside of their expanded uncertainties from those given in 11Mellau^74^ (to define reasonable expanded uncertainties for the VBOs of 11Mellau^74^, we chose the standard uncertainties reported there for the *G*_*v*_ values). Third, the computed *l*-doubling splittings^6^, listed in Tables 2 and 3, are notable for their considerable accuracy, approaching that of the measurements, and could have been used during the MARVEL analysis of this study as artificial transitions. This was not done in this study, but the computed results can be used in future high-resolution investigations of the spectroscopy of HCN isotopologues.

**Table 2:** Vibrational band origins of H^12^C^14^N with *J* = *l*_2_ = 1 and energies up to (*hc*)10 000 cm^−1^^*a*^

(*v*_1_ *v*_2_ *l*_2_ *v*_3_ *e*/*f*)	06HaTeKaPa^*a*^^17^	MARVEL this work	11Mellau^74^	21MeMaGoZo^79^
(0 1 1 0 *e*)	718.797	714.935 61(2)	714.935 65(4)^*c*^	714.935 65
(0 1 1 0 *f*)	718.812	714.950 59(2)	714.950 63(4)^*c*^	714.950 63
(0 3 1 0 *e*)	2117.260	2116.414 22(8)	2116.414 25(6)^*c*^	2116.414 25
(0 3 1 0 *f*)	2117.291	2116.445 06(8)	2116.445 09(6)^*c*^	2116.445 09
(0 1 1 1 *e*)	2816.887	2808.518 0(2)	2808.517 69(22)^*c*^	2808.517 69
(0 1 1 1 *f*)	2816.902	2808.532 0(2)	2808.532 65(22)^*c*^	2808.532 65
(0 5 1 0 *e*)	3496.817	3498.085 8(3)	3498.085 90(14)^*c*^	3498.085 90
(0 5 1 0 *f*)	3496.865	3498.133 1(3)	3498.133 56(14)^*c*^	3498.133 56
(1 1 1 0 *e*)	4007.174	4007.097 7(2)	4007.097 67(6)^*c*^	4007.097 67
(1 1 1 0 *f*)	4007.189	4007.113 5(1)	4007.112 88(6)^*c*^	4007.112 88
(0 3 1 1 *e*)	4210.518	4204.148 4(2)	4204.148 05(20)^*c*^	4204.148 05
(0 3 1 1 *f*)	4210.549	4204.179 2(2)	4204.178 74(20)^*c*^	4204.178 74
(0 7 1 0 *e*)	4865.747	4859.669(3)	4859.668 69(34)^*c*^	4859.668 69
(0 7 1 0 *f*)	4865.812	4859.735(2)	4859.734 30(34)^*c*^	4859.734 30
(0 1 1 2 *e*)	4894.823	4881.210 5(2)	4881.210 05(48)^*c*^	4881.210 05
(0 1 1 2 *f*)	4894.837	4881.225 2(2)	4881.224 99(48)^*c*^	4881.224 99
(1 3 1 0 *e*)	5369.070	5369.815(1)	5369.819 66(14)^*c*^	5369.819 66
(1 3 1 0 *f*)	5369.102	5369.851(3)	5369.851 06(14)^*c*^	5369.851 06
(0 5 1 1 *e*)	5583.938	5580.397(2)	5580.396 15(40)^*c*^	5580.396 15
(0 5 1 1 *f*)	5583.986	5580.441 5(9)	5580.443 49(40)^*c*^	5580.443 49
(1 1 1 1 *e*)	6091.508	6086.258(3)	6086.263 77(18)^*c*^	6086.263 77
(1 1 1 1 *f*)	6091.523	6086.276(1)	6086.279 11(18)^*c*^	6086.279 11
(0 9 1 0 *e*)	6218.176	−	6200.332 99(70)^*c*^	6200.332 99
(0 9 1 0 *f*)	6218.261	−	6200.417 83(70)^*c*^	6200.417 83
(0 3 1 2 *e*)	6282.881	−	6270.581 90(164)^*c*^	6270.581 90
(0 3 1 2 *f*)	6282.912	−	6270.612 38(164)^*c*^	6270.612 38
(1 5 1 0 *e*)	6709.118	−	6712.450 44(32)^*c*^	6712.450 62
(1 5 1 0 *f*)	6709.167	−	6712.499 08(32)^*c*^	6712.499 26
(0 1 1 3 *e*)	6947.413	−	−	6932.932 17
(0 1 1 3 *f*)	6947.478	−	−	6932.947 08
(0 7 1 1 *e*)	6952.437	−	−	−
(0 7 1 1 *f*)	6952.452	−	−	−
(2 1 1 0 *e*)	7193.548	7195.678 1(2)	−	7195.678 19
(2 1 1 0 *f*)	7193.563	7195.693 7(2)	−	7195.693 58
(1 3 1 1 *e*)	7448.505	−	7443.401 56(70)^*d*^	7443.401 56
(1 3 1 1 *f*)	7448.537	−	7443.433 17(70)^*d*^	7443.433 17
(0 11 1 0 *e*)	7548.596	−	−	−
(0 11 1 0 *f*)	7548.700	−	−	−
(0 5 1 2 *e*)	7649.818	−	−	−
(0 5 1 2 *f*)	7649.865	−	−	−
(1 7 1 0 *e*)	8039.015	−	8034.719 83(50)^*d*^	8034.720 29
(1 7 1 0 *f*)	8039.082	−	8034.786 93(50)^*d*^	8034.787 38
(1 1 1 2 *e*)	8155.889	−	−	8144.625 32
(1 1 1 2 *f*)	8155.905	−	−	8144.640 83
(0 9 1 1 *e*)	8295.345	−	−	−
(0 9 1 1 *f*)	8295.428	−	−	−
(0 3 1 3 *e*)	8334.189	−	−	−
(0 3 1 3 *f*)	8334.220	−	−	−
(2 3 1 0 *e*)	8518.946	−	−	8519.392 88
(2 3 1 0 *f*)	8518.867	−	−	8519.424 70
(1 5 1 1 *e*)	8782.676	−	8780.818 50(172)^*d*^	8780.818 50
(1 5 1 1 *f*)	8782.725	−	8780.867 36(172)^*d*^	8780.867 36
(0 13 1 0 *e*)	8853.559	−	−	−
(0 13 1 0 *f*)	8853.686	−	−	−
(0 1 1 4 *e*)	8989.507	−	−	−
(0 1 1 4 *f*)	8989.522	−	−	−
(0 7 1 2 *e*)	9007.461	−	−	−
(0 7 1 2 *f*)	9007.525	−	−	−
(2 1 1 1 *e*)	9262.625	9258.983(2)	−	9258.983 17
(2 1 1 1 *f*)	9262.641	9258.996 4(8)	−	9258.998 78
(1 9 1 0 *e*)	9352.239	−	−	9335.763 19
(1 9 1 0 *f*)	9352.324	−	−	9335.850 11
(1 3 1 2 *e*)	9507.069	−	−	−
(1 3 1 2 *f*)	9507.101	−	−	−
(0 11 1 1 *e*)	9620.615	−	−	−
(0 11 1 1 *f*)	9620.719	−	−	−
(0 5 1 3 *e*)	9694.325	−	−	−
(0 5 1 3 *f*)	9694.372	−	−	−
(2 5 1 0 *e*)	9819.608	−	−	−
(2 5 1 0 *f*)	9819.657	−	−	−

^*a*^ See the footnotes to Table 1.

The following observations can be made about the *J* = *l*_2_ = 0 VBOs presented in Table 1:

- The unstated origin of the ExoMol entries has been remedied in this table; the first-principles entries of the ExoMol VBOs are set in italics.
- The (2 0 0 0) VBO, for an unknown reason, is not reported in the source 21MeMaGoZo^79^.
- Because of the large discrepancy for (0 0 0 3), it is safe to assume that the (0 0 0 4) VBO, computed to be at about 8283 cm^−1^^17^, copied into the ExoMol database due to the lack of experimental/empirical results, has an error as large as 20 cm^−1^.
- The agreement between the VBOs measured in absorption and reported in 00MaMeKlWi^61^ and those of this study is significantly better than with the VBOs reported in 11Mellau^74^.
- Perhaps the largest differences between the VBOs of this study and those of 11Mellau^74^ are 0.043(30) cm^−1^ for (0 8 0 0), 0.0014(2) cm^−1^ for (0 2 0 1), where the expanded MARVEL uncertainty is given in parentheses.

As for Table 2, which contains VBOs for *J* = *l*_2_ = 1, the following additional observations can be made:

- There are small differences between the *e* and *f* states with the same (*v*_1_ *v*_2_ *l*_2_ *v*_3_) quantum numbers (*l*-type doublings, where the *f* states have the higher energy), and these differences agree very nicely with the first-principles results of 06HaTeKaPa^17^. For example, both the computed and the empirical (MARVEL) values are 0.015 cm^−1^ for the (0 1 1 0) pair (note that this high relative accuracy is not unusual; a similarly high relative accuracy has been observed for close-lying computed energies of water isotopologues that differ by one in one of the *K*_*a*_ or *K*_*c*_ values^133,134^).
- As the vibrational excitation increases, the *l*-doubling splitting also increases, and for the (2 5 1 0) pair at 9819.6 cm^−1^, the computed value is 0.049 cm^−1^, which can be compared with the empirical values of this study, 0.049(2) cm^−1^-

Similar lessons can be learned about the VBOs with *J* = *l*_2_ = 2 (see Table 3); in particular, compared to the *J* = *l*_2_ = 1 case, the *l*-doubling splittings are significantly smaller for *J* = *l*_2_ = 2, and they are always in exceedingly good agreement with the computed *l*-type doublings^17^.

**Table 3:** Vibrational band origins of H^12^C^14^N with *J* = *l*_2_ = 2 and energies up to (*hc*)10 000 cm^−1^^*a*^

(*v*_1_ *v*_2_ *l*_2_ *v*_3_ *e*/*f*)	06HaTeKaPa^17^	MARVEL this work	11Mellau^74^	21MeMaGoZo^79^
(0 2 2 0 *f*)	1441.118	1435.439 80(4)	1435.439 88(6)^*c*^	1435.439 88
(0 2 2 0 *e*)	1441.118	1435.439 90(4)	1435.439 97(6)^*c*^	1435.439 97
(0 4 2 0 *f*)	2826.240	2827.133 3(1)	2827.133 52(6)^*c*^	2827.133 52
(0 4 2 0 *e*)	2826.241	2827.133 7(1)	2827.133 81(6)^*c*^	2827.133 81
(0 2 2 1 *f*)	3536.718	3525.720(3)	3525.720 03(14)^*c*^	3525.720 03
(0 2 2 1 *e*)	3536.719	3525.719 6(2)	3525.720 13(14)^*c*^	3525.720 13
(0 6 2 0 *f*)	4200.208	4198.982 2(4)	4198.982 50(16)^*c*^	4198.982 50
(0 6 2 0 *e*)	4200.209	4198.982 2(5)	4198.983 11(16)^*c*^	4198.983 11
(1 2 2 0 *f*)	4711.051	4708.058 7(2)	4708.059 01(8)^*c*^	4708.059 01
(1 2 2 0 *e*)	4711.051	4708.059 2(2)	4708.059 11(8)^*c*^	4708.059 11
(0 4 2 1 *f*)	4916.277	−	4911.831 92(40)^*c*^	4911.831 92
(0 4 2 1 *e*)	4916.277	−	4911.832 22(40)^*c*^	4911.832 22
(0 8 2 0 *f*)	5561.807	−	5550.442 22(42)^*c*^	5550.442 22
(0 8 2 0 *e*)	5561.808	−	5550.443 28(42)^*c*^	5550.443 28
(0 2 2 2 *f*)	5611.718	5594.851 1(3)	5594.851 13(38)^*c*^	5594.851 13
(0 2 2 2 *e*)	5611.718	5594.851 4(3)	5594.851 23(38)^*c*^	5594.851 23
(1 4 2 0 *f*)	6057.816	−	6060.818 98(14)^*c*^	6060.818 98
(1 4 2 0 *e*)	6057.816	−	6060.819 28(14)^*c*^	6060.819 28
(0 6 2 1 *f*)	6284.218	−	6278.422 25(272)^*c*^	6278.422 25
(0 6 2 1 *e*)	6284.218	−	6278.422 85(272)^*c*^	6278.422 85
(1 2 2 1 *f*)	6792.993	6784.151(7)	6784.171 92(20)^*c*^	6784.17 192
(1 2 2 1 *e*)	6792.993	6784.166(7)	6784.172 02(20)^*c*^	6784.172 02
(0 10 2 0 *f*)	6904.058	−	−	6880.433 72
(0 10 2 0 *e*)	6904.059	−	−	6880.435 42
(0 4 2 2 *f*)	6985.156	−	−	−
(0 4 2 2 *e*)	6985.156	−	−	−
(1 6 2 0 *f*)	7392.476	−	7393.466 64(24)^*d*^	7393.466 64
(1 6 2 0 *e*)	7392.477	−	7393.467 29(24)^*d*^	7393.467 29
(0 8 2 1 *f*)	7640.941	−	−	7642.772 16
(0 8 2 1 *e*)	7640.942	−	−	7642.772 26
(0 2 2 3 *f*)	7665.927	−	−	−
(0 2 2 3 *e*)	7665.928	−	−	−
(2 2 2 0 *f*)	7879.026	7876.853 3(2)	−	7876.853 64
(2 2 2 0 *e*)	7879.026	7876.853 8(2)	−	7876.853 75
(1 4 2 1 *f*)	8134.193	−	8131.572 29(78)^*d*^	8131.572 29
(1 4 2 1 *e*)	8134.193	−	8131.572 61(78)^*d*^	8131.572 61
(0 12 2 0 *f*)	8222.499	−	−	−
(0 12 2 0 *e*)	8222.502	−	−	−
(0 6 2 2 *f*)	8346.775	−	−	−
(0 6 2 2 *e*)	8346.776	−	−	−
(1 8 2 0 *f*)	8715.116	−	8705.444 86(150)^*d*^	8705.444 86
(1 8 2 0 *e*)	8715.118	−	8705.446 00(150)^*d*^	8705.446 00
(1 2 2 2 *f*)	8854.529	−	−	−
(1 2 2 2 *e*)	8854.529	−	−	−
(0 10 2 1 *f*)	8978.628	−	−	−
(0 10 2 1 *e*)	8978.630	−	−	−
(0 4 2 3 *f*)	9032.722	−	−	−
(0 4 2 3 *e*)	9032.722	−	−	−
(2 4 2 0 *f*)	9187.375	−	−	9190.517 97
(2 4 2 0 *e*)	9187.376	−	−	9190.518 29
(1 6 2 1 *f*)	9463.173	−	9459.163 98(150)^*d*^	−
(1 6 2 1 *e*)	9463.173	−	9459.164 64(150)^*d*^	−
(0 14 2 0 *f*)	9514.042	−	−	−
(0 14 2 0 *e*)	9514.046	−	−	−
(0 2 2 4 *f*)	9698.071	−	−	−
(0 2 2 4 *e*)	9698.072	−	−	−
(0 8 2 2 *f*)	9699.163	−	−	−
(0 8 2 2 *e*)	9699.163	−	−	−
(2 2 2 1 *f*)	9945.941	−	−	9937.501 80
(2 2 2 1 *e*)	9945.941	−	−	9937.501 90

^*a*^ See the footnotes to Table 1.

#### The 06HaTeKaPa^17^ first-principles computed rovibrational dataset

**Fig. 5:**
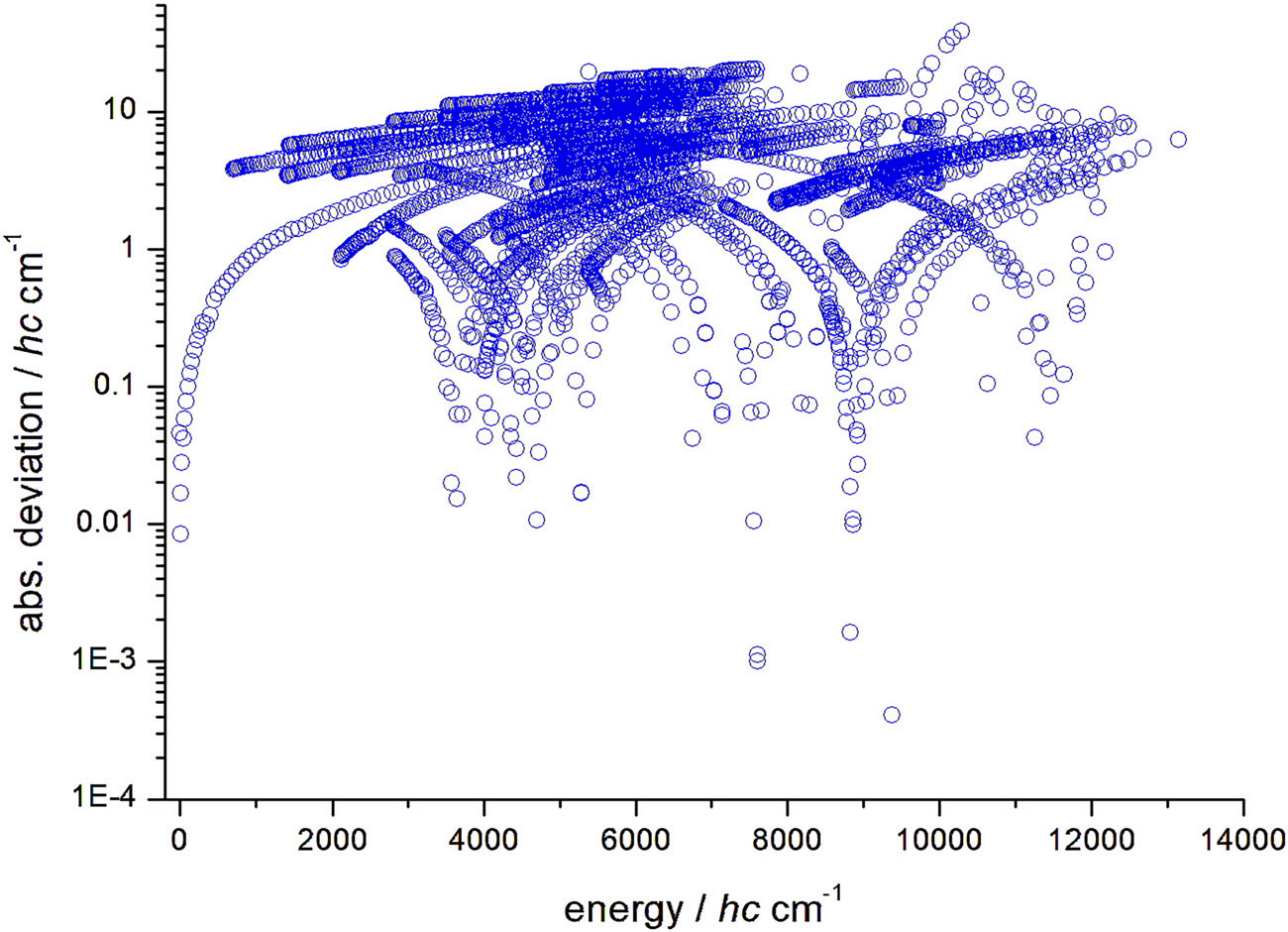
Comparison between the empirical rovibrational energies of the present H^12^C^14^N dataset with those of 06HaTeKaPa^17^, containing mostly first-principles computed results.

The first-principles computed 06HaTeKaPa^17^ dataset, which can be downloaded from the website https://cdsarc.cds.unistra.fr/ftp/VI/121/, contains 18 723 assigned H^12^C^14^N rovibrational energy levels, up to *J* = 60, in the (*hc*)0 − 14 740 cm^−1^ energy range. Figure 5 shows the absolute deviations of the rovibrational energy levels of H^12^C^14^N between the empirical (MARVEL) and the 06HaTeKaPa datasets when this comparison can be made. The root-mean-square deviation of these two datasets is (*hc*)7.6 cm^−1^, indicating reasonable agreement when accurate empirical energies are compared with first-principles ones.

#### The 11Mellau^74^ “experimental” rovibrational dataset

The source 11Mellau^74^ provides two sets of so-called “experimental” rovibrational energies for H^12^C^14^N (following the terminology of the present paper, these energies could also be referred to as empirical). First, in Tables IV–XIX of ref. ^74^, there is a complete list of a total of 3840 rovibrational energy levels between (*hc*)2.956 43 and (*hc*)6887.829 37 cm^−1^ (relative to the ground vibrational state). Second, in the Supplementary Material to this paper, there are 11,070 rovibrational eigenenergies reported, extending up to *J* = 90 for the first 123 rovibrational progressions.

A detailed comparison between the complete lists of Tables IV–XIX of ref. ^74^ up to 6888 cm^−1^ and the Supplementary Material reveals that the *J* = 2 *e*/*f* pair of the (0 10 2 0) energy levels is presented in the article but not in the Supplementary Material.

#### The ExoMol^18^ rovibrational dataset

**Fig. 6:**
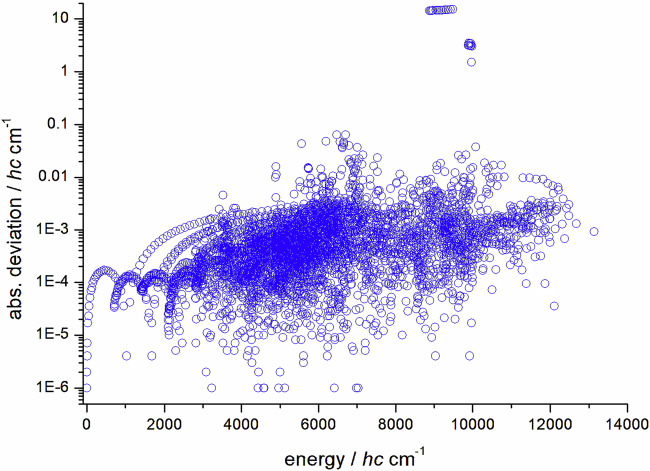
Comparison between the empirical rovibrational energies of the present H^12^C^14^N dataset with those of ExoMol^18^.

The H^12^C^14^N ExoMol rovibrational line list^18^ is a mixture of energy levels taken from 11Mellau^74^ and other experimental sources available at the time, along with the pure ab initio results of 06HaTeKaPa^17^ for HCN, plus a similar treatment for HNC. Figure 6 of this study considers only the HCN levels. Thus, it is not surprising that in this figure the absolute deviations between the energy values in the ExoMol dataset and the empirical (MARVEL) energy levels of this study are very similar, as the majority of the energy levels of the ExoMol dataset below (*hc*)10 000 cm^−1^ and the 21MeMaGoZo^79^ dataset use exactly the same set of eigenenergies provided by 11Mellau^74^.

A characteristic of the 14BaStHiPo^18^ ExoMol line list is that it does not distinguish between empirical and ab initio energy levels. This means that it is not possible to determine whether an energy level listed in ExoMol is of purely theoretical or empirical/experimental origin. This issue has been addressed in more recent ExoMol datasets, e.g., ref. ^135^, which explicitly provide the provenance of the individual energy levels.

#### The MOMeNT-90^79^ dataset

An H^12^C^14^N-only line list called MOMeNT-90, covering the spectral region 0 − 7500 cm^−1^, was provided by 21MeMaGoZo^79^. The MOMeNT-90 line list is a combination of more than 133 000 line centers obtained strictly from experimentally derived energy levels of Mellau^74–76^ and variationally computed line intensities. The line intensities are based on wavefunctions generated using an HCN potential energy surface fitted to the experimental energy levels^2^ and an updated ab initio dipole moment surface^136^.

**Fig. 7:**
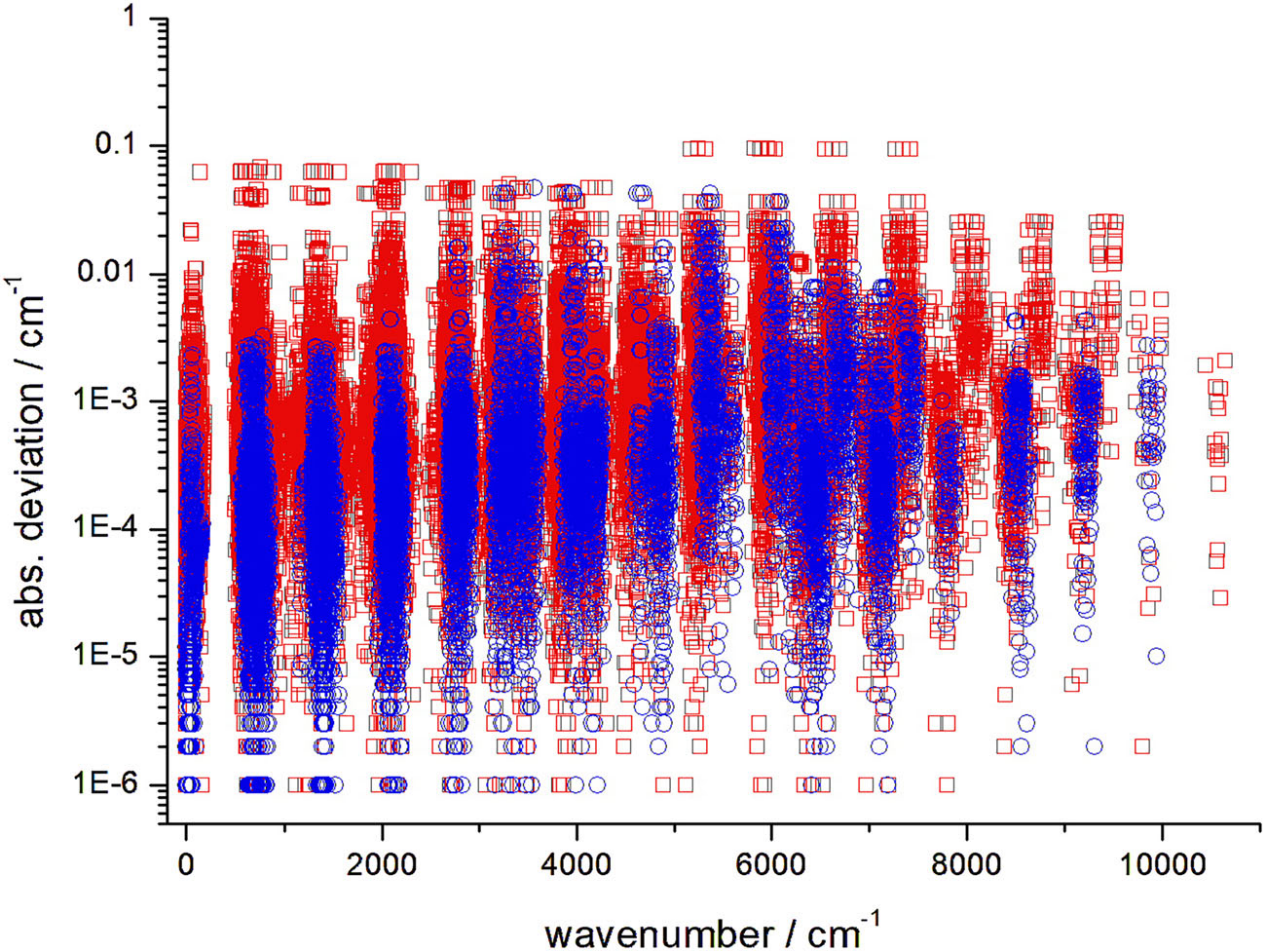
Comparison between the empirical line list of the present H^12^C^14^N dataset and the MOMeNT-90 positions^79^. The blue circles represent lines with an intensity larger than 1 × 10^−26^ cm molecule^−1^, while the red squares show lines weaker than this.

Figure 7 shows the absolute deviations between the experimental line centers of MOMeNT-90 and the line positions obtained from the empirical (MARVEL) energy levels of this study. The blue circles represent strong lines (their intensity, as given in the MOMeNT-90 line list, is larger than 1 × 10^−26^ cm molecule^−1^), while the red squares belong to lines weaker than this. As can be observed in Fig. 7, the agreement of the great majority of the strong (blue) lines is significantly better than 0.01 cm^−1^ even up to wavenumber values of 10 000 cm^−1^. The differences between the weak MOMeNT-90 and MARVEL line positions can be somewhat larger; though they never go beyond 0.1 cm^−1^.

**Fig. 8:**
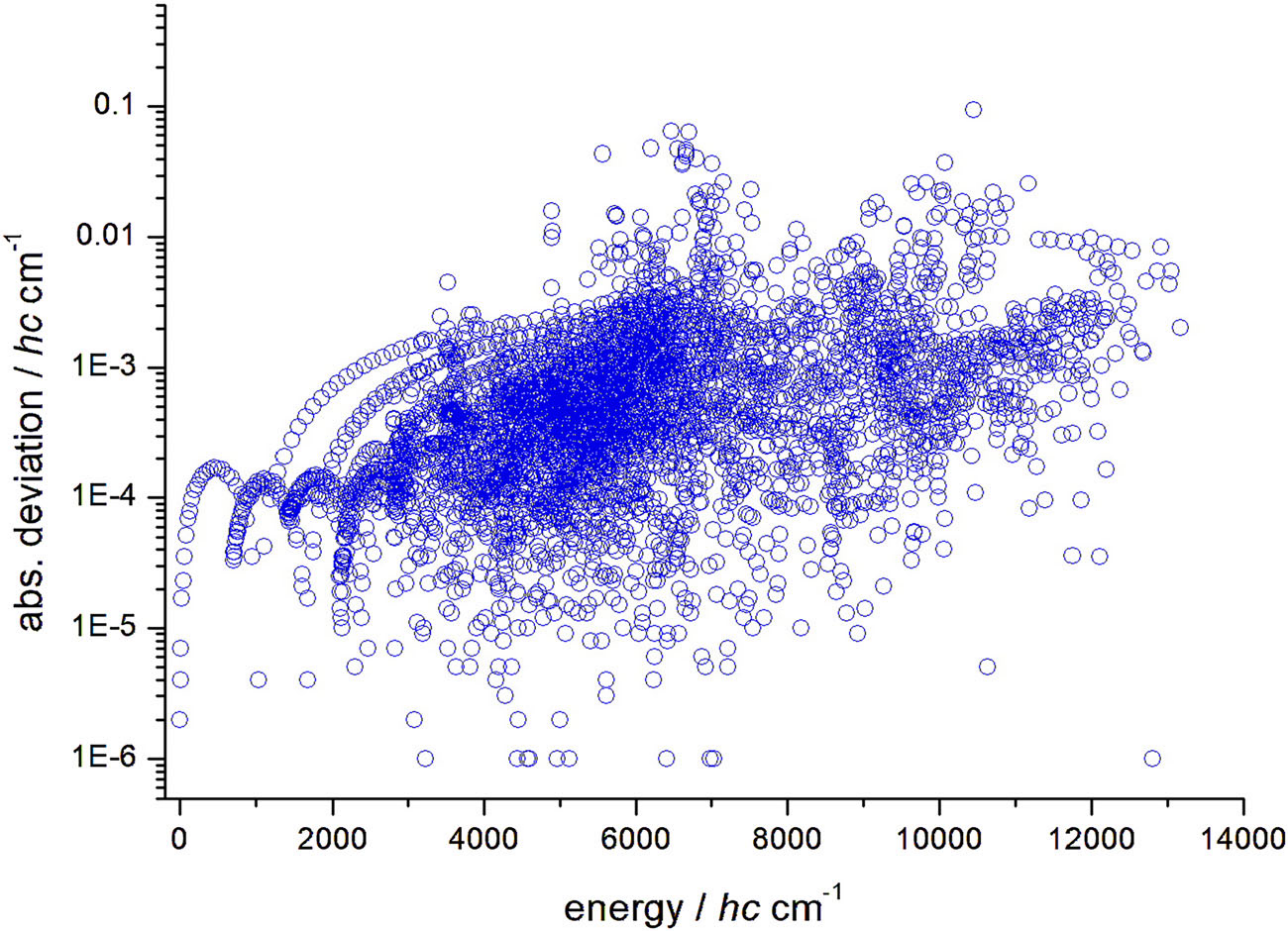
Comparison between the empirical energy list of the present H^12^C^14^N dataset and the MOMeNT-90 energies^79^.

Figure 8 shows the absolute deviations between the experimental energy values of MOMeNT-90 and the empirical (MARVEL) energy levels of this study. It can be seen that the largest difference between the MARVEL and MOMeNT-90 energies is less than (*hc*)0.1 cm^−1^.

#### The HITRAN2020^129^ database

Of the 128 236 line positions the canonical HITRAN2020^129^ database contains for H^12^C^14^N, only 112 834 have full assignments. During the present study, no attempt was made to provide assignments for the 15 402 HITRAN2020 lines with incomplete assignments.

**Fig. 9:**
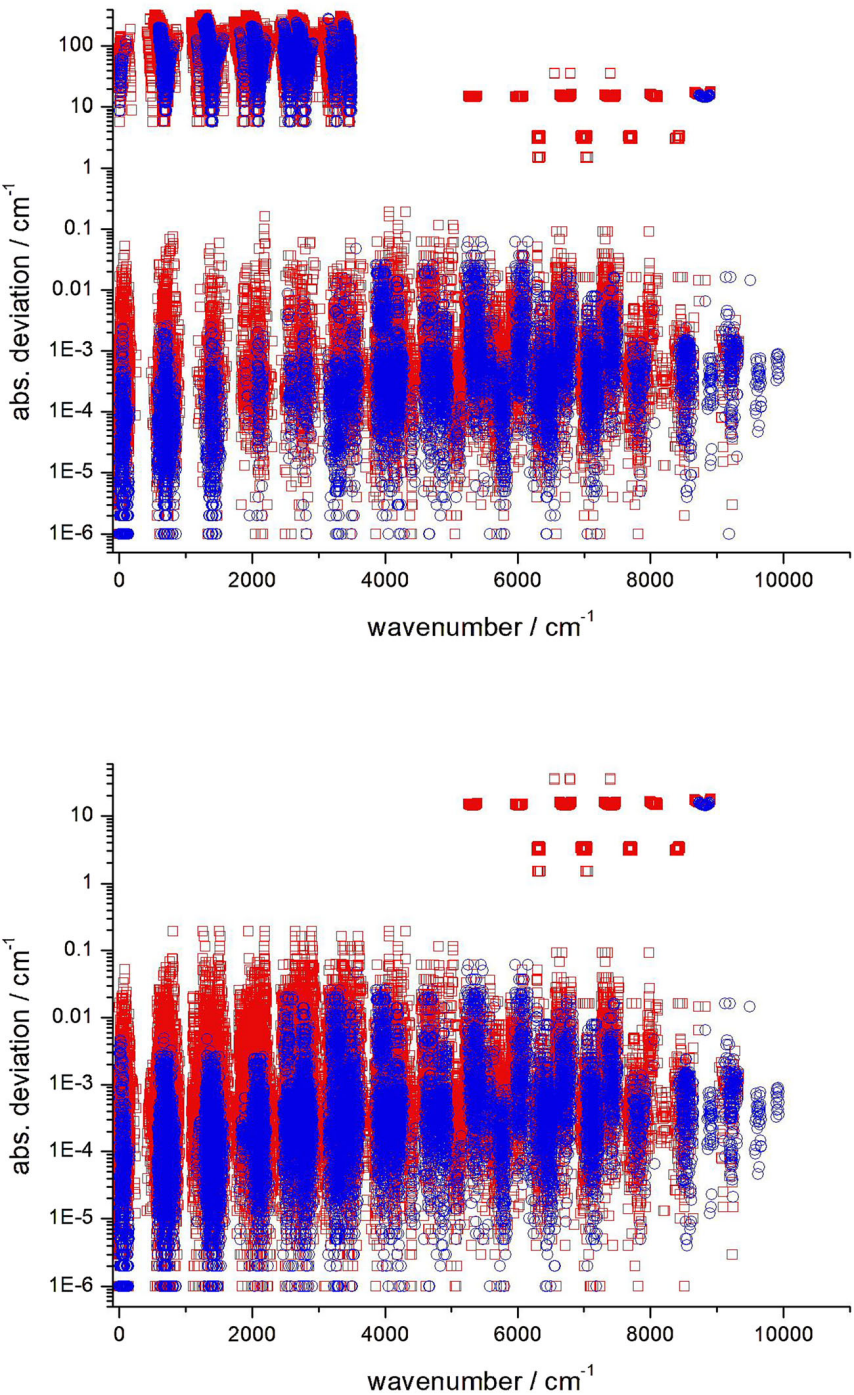
Comparison between the empirical transitions of H^12^C^14^N determined in this study and those of HITRAN2020^129^. To make the important discrepancies between the two datasets more transparent, the blue circles represent lines with intensities larger than 1 × 10^−26^ cm molecule^−1^ (these are deemed to be the important discrepancies), while the red squares show lines weaker than this. Top panel: deviations of the empirical (MARVEL) results from the original entries of the HITRAN2020 database. Bottom panel: the deviations from the HITRAN2020 database after the HITRAN2020 transitions were corrected according to Sec. “The HITRAN2020 database”.

The HITRAN2020 database took more than 95% of its transition entries from the source 14BaStHiPo^18^. The energy level set 14BaStHiPo was created by merging the following two sets of energy levels: the less accurate first-principles set 06HaTeKaPa and the accurate “experimental” set MOMeNT-90. Therefore, it was expected that the comparison of the present MARVEL and the HITRAN2020 datasets would result in a figure that was a combination of Figs. 5 and 7. The top panel of Fig. 9 shows the actual absolute deviations between the HITRAN2020 line centers and the line positions obtained from the empirical (MARVEL) energy levels of this study. To make the important discrepancies between the two datasets more transparent, in Fig. 9 the blue circles represent the relatively strong lines (whose room-temperature intensity, given in the HITRAN2020 database, is larger than 1 × 10^−26^ cm molecule^−1^, these are the discrepancies deemed to be the most important for the modeling community), while the red squares belong to lines weaker than this.

As seen in the top panel of Fig. 9a there are many cases, more than 40 000, where the difference between the HITRAN2020 and the empirical (MARVEL) line positions is larger than 1.0 cm^−1^, although for the majority of the lines the agreement, as expected, is better than 0.1 cm^−1^, and (b) the large differences appear to be independent of the intensity. Since the majority of the HITRAN transitions originate from the source 14BaStHiPo^18^, we performed a systematic comparison between these two line lists. Let us consider two lines to be identical if their full assignments are the same, their intensities differ by a maximum factor of 2, and the difference in their wavenumbers is less than 0.005 cm^−1^. The results of this comparison are as follows:

- Of the 112 834 fully assigned HITRAN2020 transitions, in 67 937 cases we found “perfect” matches with the entries of the 14BaStHiPo^18^ line list.
- In 44 858 cases, the wavenumbers and the intensities of the two lines compared fulfilled our matching requirements, and the assignments of the lower energy states were the same, but the upper rotational quantum number ($J^{\prime}$) was different. The HITRAN2020 database uses the standard P, Q, and R notation to describe the rotational transitions, and for some unknown reason, incorrect rotational labels (P, Q, and R) were assigned to these lines. For example, (a) the ‘R7e’ line at 0.417 87 cm^−1^ should be the ‘Q7e’ line and (b) the ‘Q1e’ line at 5.870 96 cm^−1^ is in fact the ‘R1e’ line. Based on the present global MARVEL analysis, it is straightforward to correct these incorrect HITRAN2020 entries.
- In the case of 39 HITRAN2020 transitions, we did not find a match in the source 14BaStHiPo^18^. These incorrect lines are listed in Table 4, and we are providing comments that attempt to explain the origin of the present situation.

Fixing all the problems found and indicated above resulted in the bottom panel of Fig. 9. It shows that (a) all the large “errors” below 5500 cm^−1^ disappear with the use of the correct rotational labels, and (b) there are no changes in the outliers above 5500 cm^−1^, which is fine, as the deviations between 1 and 10 cm^−1^ above 5500 cm^−1^ are due to first-principles entries in HITRAN2020. Since the present MARVEL analysis provides supposedly improved estimates for these lines (of low intensity, less than 1 × 10^−26^ cm molecule^−1^, below 9000 cm^−1^), they could be considered for replacement in a future edition of the HITRAN database. This line list is given in the Supplementary Information.

**Table 4:** Line center positions in the HITRAN2020 compilation^129^ which could not be validated during the present study

HITRAN2020 wavenumber/cm^−1^	full assignment	comment
2.971 652	(1 0 2 0 0 *e*) − (0 0 2 0 0 *e*)	a
2.971 726	(1 0 2 0 0 *e*) − (0 0 2 0 0 *e*)	a
77.233 582	(26 0 2 2 0 *e*) − (25 0 2 2 0 *e*)	b
463.162 870	(7 0 1 1 0 *e*) − (6 0 0 0 0 *e*)	c
463.309 580	(8 0 1 1 0 *e*) − (7 0 0 0 0 *e*)	c
463.477 140	(9 0 1 1 0 *e*) − (8 0 0 0 0 *e*)	c
463.665 510	(10 0 1 1 0 *e*) − (9 0 0 0 0 *e*)	c
463.874 640	(11 0 1 1 0 *e*) − (10 0 0 0 0 *e*)	c
719.406 730	(24 0 3 3 0 *e*) − (24 0 2 2 0 *f*)	c
721.830 760	(33 0 3 3 0 *e*) − (33 0 2 2 0 *f*)	c
727.897 350	(47 0 1 1 0 *f*) − (47 0 0 0 0 *e*)	c
1458.695 649	(18 0 3 1 0 *f*) − (17 0 1 1 0 *f*)	c
2522.349 652	(26 0 1 1 0 *e*) − (26 0 5 5 0 *f*)	c
2674.348 972	(25 0 1 1 0 *f*) − (24 0 5 5 0 *f*)	c
2678.761 302	(25 0 1 1 0 *e*) − (25 0 5 5 0 *f*)	c
2683.217 832	(26 0 1 1 0 *e*) − (26 0 5 5 0 *f*)	c
2687.717 272	(28 0 1 1 0 *f*) − (27 0 5 5 0 *f*)	c
2692.258 332	(29 0 1 1 0 *f*) − (28 0 5 5 0 *f*)	c
3241.361 852	(31 0 1 1 0 *f*) − (30 0 4 4 0 *f*)	c
3242.629 032	(29 0 1 1 0 *e*) − (29 0 4 4 0 *f*)	c
3243.945 182	(29 0 1 1 0 *f*) − (28 0 4 4 0 *f*)	c
3245.311 262	(28 0 1 1 0 *f*) − (27 0 4 4 0 *f*)	c
3246.728 272	(26 0 1 1 0 *e*) − (26 0 4 4 0 *f*)	c
3262.287 350	(45 1 2 2 0 *e*) − (45 0 2 2 0 *f*)	d
3324.090 162	(26 0 1 1 0 *e*) − (25 0 4 4 0 *e*)	c
3325.633 042	(27 0 1 1 0 *e*) − (26 0 4 4 0 *e*)	c
3327.223 812	(28 0 1 1 0 *e*) − (27 0 4 4 0 *e*)	c
3328.860 972	(29 0 1 1 0 *e*) − (28 0 4 4 0 *e*)	c
3330.542 942	(30 0 1 1 0 *e*) − (29 0 4 4 0 *e*)	c
3337.579 722	(18 0 4 2 0 *f*) − (17 0 6 6 0 *f*)	c
3341.894 792	(19 0 4 2 0 *f*) − (18 0 6 6 0 *f*)	c
3381.404 750	(43 1 2 2 0 *e*) − (42 0 2 2 0 *e*)	c
3385.491 041	(45 1 2 2 0 *e*) − (44 0 2 2 0 *e*)	c
3387.501 285	(46 1 2 2 0 *e*) − (45 0 2 2 0 *e*)	c
3398.516 822	(24 0 1 1 0 *e*) − (24 0 4 4 0 *f*)	c
3403.032 382	(26 0 1 1 0 *f*) − (25 0 4 4 0 *f*)	c
3407.596 452	(27 0 1 1 0 *f*) − (26 0 4 4 0 *f*)	c
3412.207 902	(27 0 1 1 0 *e*) − (27 0 4 4 0 *f*)	c
3416.865 612	(27 0 1 1 0 *f*) − (28 0 4 4 0 *f*)	c

^*a*^ Probably a hyperfine transition; according to HITRAN2020, the source of this line is 03ZeAmAhBr^67^, but we could not locate this transition in it.

^*b*^ Duplicate assignment; one of the lines with this assignment should be deleted.

^*c*^ This wavenumber can not be found in 14BaStHiPo^18^ and the full assignment is also incorrect; it is our recommendation that this line should be deleted from the HITRAN2020 database.

^*d*^ According to HITRAN2020, the source of this line is 00MaMeKlWi^61^, but we could not locate it.

Since HITRAN2020 seemingly contains numerous errors in the transition wavenumbers for H^12^C^14^N, we have decided to create a corrected version of the HITRAN2020 line list. The format of the published file is identical to the original HITRAN2020 file format, but we have added an extra column indicating corrections that have been made or are to be made. The replacement of incorrect rotation notation is indicated by the comment ‘old_notation  → new_notation’. For example, ‘Q  → R’ means that we replaced the descriptor Q with R. Our suggested corrections include rows marked for deletion (‘DEL’) or wavenumber replacement (‘WN new_value’).

## Conclusions

This paper describes a comprehensive, critical, line-by-line analysis of all available measured and assigned rovibrational transitions corresponding to the ground electronic state of the parent isotopologue, H^12^C^14^N, of hydrogen cyanide. Our detailed and careful joint analysis of the measured transitions utilized the MARVEL algorithm^102–104^ and the latest version of the associated code^137^.

In total, 23 225/14 728 non-unique/unique transitions, almost exclusively experimentally measured ones, were collected for H^12^C^14^N from the literature, covering the wavenumber range of 0 − 13 000 cm^−1^, and the transitions dataset is characterized by ${J}_{\max }=66$ and ${P}_{\max }=21$, where *J* is the rotational quantum number, and *P* is the polyad number *P* = 5*v*_1_ + *v*_2_ + 3*v*_3_. We were able to validate 22 647 transitions (note that transitions within floating components cannot be validated using the spectroscopic-network-based MARVEL procedure). This set of validated transitions yields 5564 empirical (MARVEL) rovibrational energy levels with dependable, experimentally-based uncertainties. The average expanded uncertainty of the empirical rovibrational energy levels of H^12^C^14^N, derived from the experimental expanded uncertainties of the transitions, is (*hc*)0.006 cm^−1^.

Comparison of the empirical energy levels and the validated transitions of this study with their counterparts in the HITRAN2020^129^, ExoMol^18,128^, and MOMeNT-90^79^ datasets reveals a good overall agreement, as expected. Nevertheless, these comparisons highlight a couple of issues with these datasets, especially with the labels published in HITRAN2020.

The empirical energy levels derived in this study can be used to improve the accuracy of the ExoMol dataset for HCN by a minor amount. Note, in this respect, that the most recent ExoMol line list^18^ has been extensively used in explorations of exoplanets during both low-^94,95^ and high-resolution^91^ studies. Furthermore, it was used successfully very recently to analyze new sub-millimeter HCN laser lines in carbon-rich evolved stars^96^. As the ExoMol line list^18^ covers both HCN and HNC, a study similar to the present one would also be required for the HNC isomer. However, given the fact that it is almost only the self-consistent set of studies of Mellau^72,73,75,77^ that adequately addressed the spectroscopy of HNC, it is unlikely that a MARVEL study would yield significant new information.

## Methods

### Theory

#### Notation, quantum numbers, and selection rules

Of the three vibrational fundamentals of triatomic HCN, the first, *ν*_1_, of *Σ*^+^ symmetry, corresponds to the CH stretch (with a wavenumber of 3311.48 cm^−1^), *ν*_2_ is the degenerate bending fundamental (at 713.46 cm^−1^) of *Π* symmetry, while *ν*_3_ is the CN stretch fundamental (at 2096.85 cm^−1^), of *Σ*^+^ symmetry^70^. According to the selection rules of quantum mechanics, transitions with *Δv*_*i*_ = ± 1, ± 2, ± 3, etc., are all allowed, where there is a direct one-to-one correspondence between the vibrational modes and the vibrational quantum numbers, *ν*_*i*_ and *v*_*i*_, respectively.

Table 5 summarizes the rovibrational quantum numbers and the associated state descriptors of H^12^C^14^N used in this study. The angular momentum associated with the degenerate *ν*_2_ bending mode of vibration, *l*_2_, can take on values of *v*_2_, *v*_2_ − 2, (1/0). *l*-type doubling occurs for all values of *l*_2_ greater than one^138^. Note that some older articles use ‘*c*’ and ‘*d*’ instead of ‘*e*’ and ‘*f*’ for the rotationless parity, see ref. ^139^.

**Table 5:** Rovibrational quantum numbers and associated state descriptors used in this study for H^12^C^14^N

Descriptor	Description
*J*	End-over-end rotational quantum number
*v*_1_	C–H stretching mode
*v*_2_	Doubly degenerate bending mode
*l*_2_	Vibrational angular momentum associated with the *ν*_2_ mode
*v*_3_	CN stretching mode
*P*	polyad number, *P* = 5*v*_1_ + *v*_2_ + 3*v*_3_
*e*/*f*	Rotationless parity

In spectroscopy, it has been common practice to use polyad numbers (*P*) to denote strongly interacting groups of vibrational states. Though *P* is not a true quantum number, it behaves like one. The approximate 4.5:1:3 relation of the three vibrational fundamentals of H^12^C^14^N suggests the following definition for the polyad number: *P* = 5*v*_1_ + *v*_2_ + 3*v*_3_, implying that *P* takes consecutive non-negative integer values starting from zero. This definition is used in this article. Although *P* is not directly relevant to the MARVEL analysis of the measured transitions, it allows one to perform certain checks on the input transitions and the output energy levels.

The rovibrational transitions, following the standard selection rules *ΔJ* = 0,  ± 1, can be grouped into three branches: the P, Q, and R branches have *ΔJ*  = − 1, 0, and +1, respectively. For the P and R branches, the initial and final states must have the same rotationless parity. For the Q branch, the initial and final states must change the rotationless parity. When *l*_2_ = 0, *J* can only have *e* rotationless parity, and only *ΔJ* = ± 1 is allowed. If *Δl*_2_ = ± 1, then *ΔJ* = ± 1 or 0 can occur, resulting in a strong Q-branch alongside the P and R branches. If *Δl*_2_ = 0, but *l*_2_ ≠ 0 for both states, the Q branches are generally weaker than the P and R branches.

Some spectroscopic studies, for example, 02AhLeTaWi^63^, achieved a resolution such that they were able to report the positions of hyperfine lines. In cases when the authors report hyperfine-free rotational frequencies, this is the information that has been entered into the MARVEL input file (see the Supplementary Information for this paper).

#### MARVEL

Measured Active Rotational-Vibrational Energy Levels (MARVEL)^102–107,137,140^ is a powerful tool; the related MARVEL code has been used in this study for two main purposes: (a) to efficiently analyze and subsequently validate all the measured line-center positions and the related assignments available for H^12^C^14^N, and (b) to determine empirical energy levels from the validated set of experimental transitions.

MARVEL operates by constructing a so-called spectroscopic network (SN)^108,109^ from the complete set of measured transitions. In SNs, the energy levels form the set of vertices, and the transitions between them form the set of edges. For a successful MARVEL analysis, it is essential that each SN transition has an accurate line-center position with an associated physically meaningful uncertainty (the latter is especially important). The rovibrational transitions used in this study are characterized by expanded uncertainties *U* with a coverage factor of *k* = 2, roughly corresponding to a 95 % confidence interval. It is an additional requirement that each transition’s upper and lower energy levels must be uniquely labeled.

In the input file to MARVEL, each transition is characterized by (a) a line position (in units indicated in the ‘segment’ file associated with the input file), (b) an initial and an adjusted, expanded (two-sigma) uncertainty for the line position (the adjusted uncertainty is the one that ensures that the final set of input transitions is self-consistent), (c) the rovibrational assignments of the upper and lower states (see Sec. “Notation, quantum numbers, and selection rules” for a description of the labels of the quantum states and the selection rules), and (d) a line tag, a metadata string providing a unique identifier for the source of the transition (for multi-author publications, each line tag starts with the last two digits of the year of publication, augmented with the first two characters of the last names of the authors, up to the first four authors), followed by a sequence number for each line.

In those cases where the experimental data are incomplete, the SN built from the measured transitions may become fragmented, leaving some energy levels isolated from the root, the lowest-energy quantum state of the molecular system (meaning that there is no path connecting them). The SN of H^12^C^14^N contains a single principal component, which is a connected set of states and transitions originating from the root, as there is only one nuclear spin isomer of H^12^C^14^N^105,130^. It is often feasible to connect the isolated, “floating” components of the SN to the principal component(s) using accurate empirical data (such as those derived from effective Hamiltonian fits). This has been done during the present study. Note that, in some cases, this connection can also be established by exploiting known quasi-degeneracies of certain energy levels^115,133,134^, but this has not been done during this study.

Unlike in cases of advanced quantum-chemical models, which rely on solving the complete nuclear Schrödinger equation using an approximate potential energy surface^141,142^, the accuracy of the empirical MARVEL energy levels is not constrained by assumptions beyond the validity of the simple Ritz principle^143^ and the accuracy of the measured line-center positions. Similarly, accidental resonances between rovibrational states require the use of special measures within the framework of effective Hamiltonians, but no adjustment is needed to the MARVEL algorithm. Thus, the MARVEL approach facilitates the model-free transfer of the usually exceedingly high accuracy of the measured transitions to the empirical energy levels. At the same time, it is important to acknowledge that MARVEL can also accept transitions that violate standard quantum mechanical spectroscopic selection rules or are otherwise incorrect, as long as they do not cause conflict with the other entries of the assembled dataset.

The MARVEL procedure for generating empirical energy levels from measured transitions begins by collecting all relevant high-resolution spectroscopic rovibrational data. Once a dataset of experimental rovibrational transitions is compiled, it is processed using the MARVEL algorithm, which ensures self-consistency by identifying and flagging errors and inconsistencies, such as typographical mistakes or misassignments. In some cases, conflicts cannot be resolved based on the available information; as a result, these lines must be excluded from the final MARVEL analysis. The wavenumber entries of these lines are marked with a negative sign in the MARVEL input file.

The final set of empirical rovibrational energy levels was used to generate a large number of rovibrational transitions, which we could compare with their counterparts in several available datasets, such as the first-principles list of Harris et al.^17^, the “experimental” levels of Mellau^74–76^, the line list MOMeNT-90^79^, the mixed empirical and first-principles ExoMol^18,128^ line list, and the canonical line list of HITRAN2020^129^. Details about these comparisons and the lessons that can be learned from them are discussed in Sec. “Comparison with other datasets”.

### Data sources

Certain characteristics of the literature sources containing the measured rovibrational transitions of H^12^C^14^N are summarized in Table 6. Further characteristics of some of these sources are discussed in the next subsections.

**Table 6:** Data sources used in this study for the analysis of the rovibrational energy-level structure of the hydrogen cyanide isotopologue H^12^C^14^N

Source	Range/cm^−1^	*A*/*F*/*V*/*D*	*ΔU*^CSU^/cm^−1^	*ΔU*^MSU^/cm^−1^
25CDMS^145^	0.01 − 192.65	699/0/697/2	1.2 × 10^−5^	1.2 × 10^−5^
56Yarmus^26^	0.01 − 0.22	5/0/5/0	3.5 × 10^−7^	5.2 × 10^−7^
70RaKu^37^	0.01 − 0.01	1/0/1/0	2.7 × 10^−8^	2.7 × 10^−8^
03ZeAmAhBr^67^	0.04 − 65.67	202/0/198/4	6.9 × 10^−7^	7.8 × 10^−7^
84FlDrCoHu^47^	0.15 − 0.54	5/0/5/0	1.7 × 10^−7^	1.7 × 10^−7^
61Torring^30^	0.22 − 0.86	12/0/12/0	2.3 × 10^−6^	2.6 × 10^−6^
67MaLi^34^	0.28 − 3.13	19/0/19/0	3.3 × 10^−6^	3.3 × 10^−6^
54Westerka^23^	0.31 − 1.17	6/0/6/0	6.7 × 10^−6^	1.2 × 10^−5^
03ThMuLeBr^66^	1.79 − 65.23	28/0/28/0	6.7 × 10^−7^	6.7 × 10^−7^
77LuHe^44^	2.92 − 11.98	48/0/44/4	1.7 × 10^−6^	2.2 × 10^−6^
02AhLeTaWi^63^	2.96 − 64.92	20/0/20/0	7.1 × 10^−7^	7.3 × 10^−7^
74Maki^42^	2.96 − 8.87	3/0/3/0	6.1 × 10^−6^	6.1 × 10^−6^
71WiMaJo^38^	2.97 − 5.94	4/0/3/1	7.1 × 10^−6^	7.1 × 10^−6^
90SaYa^55^	5.91 − 6408.68	48/0/48/0	3.8 × 10^−4^	5.0 × 10^−4^
00MaLeAhBe^60^	8.87 − 64.92	18/0/18/0	3.4 × 10^−6^	3.8 × 10^−6^
96MaQuKlMe^58^	26.84 − 139.08	21/4/17/0	9.9 × 10^−3^	6.8 × 10^−3^
67HoJaRaFr^33^	29.71 − 29.71	1/0/1/0	6.7 × 10^−6^	6.7 × 10^−6^
00MaMeKlWi^61^	528.02 − 9688.21	13 292/83/13 209/0	5.1 × 10^−4^	5.3 × 10^−4^
60BrHoNiNa^27^	594.11 − 1522.07	234/0/221/13	1.0 × 10^−2^	1.7 × 10^−2^
89DuGa^53^	606.88 − 814.68	442/0/441/1	6.8 × 10^−4^	8.9 × 10^−4^
73WaOv^40^	623.57 − 1523.94	588/0/588/0	9.4 × 10^−3^	1.2 × 10^−2^
05DeBeSmRi^69^	629.30 − 797.25	54/0/54/0	3.1 × 10^−5^	8.2 × 10^−5^
72YiRa^39^	629.30 − 797.25	216/0/183/33	1.2 × 10^−3^	4.2 × 10^−3^
08SmRiBlSa^71^	632.24 − 808.89	247/0/247/0	2.0 × 10^−4^	2.3 × 10^−4^
88HiJoHo^51^	646.97 − 785.58	53/0/53/0	3.0 × 10^−4^	3.0 × 10^−4^
04DeBeSmRi^68^	1324.23 − 1497.79	146/0/146/0	1.3 × 10^−4^	1.7 × 10^−4^
87ChKwKu^50^	1999.69 − 2234.84	393/0/393/0	1.3 × 10^−3^	1.4 × 10^−3^
64MaBl^31^	1999.71 − 3986.67	422/0/418/4	1.0 × 10^−2^	1.6 × 10^−2^
00PiGu^59^	2452.78 − 3403.53	936/3/837/96	1.0 × 10^−3^	1.5 × 10^−3^
70MaOlSa^36^	2690.21 − 2903.93	344/0/341/3	6.0 × 10^−3^	6.1 × 10^−3^
86ChTiKu^49^	3170.29 − 3418.05	411/0/411/0	4.6 × 10^−4^	7.5 × 10^−4^
61RaEaRaWi^29^	3174.04 − 3416.04	67/0/67/0	1.0 × 10^−3^	1.9 × 10^−3^
03DeBeSmRi^64^	3201.59 − 3399.17	174/0/174/0	2.2 × 10^−4^	4.4 × 10^−4^
80GeGo^46^	3234.94 − 3378.44	49/0/49/0	3.0 × 10^−3^	3.2 × 10^−3^
85KyChElDa^48^	3284.13 − 3339.88	19/0/19/0	6.7 × 10^−4^	8.1 × 10^−4^
76MaSa^43^	4836.65 − 6097.51	153/0/153/0	7.0 × 10^−3^	1.6 × 10^−2^
08MeWiWi^70^	6122.51 − 6816.21	3034/270/2764/0	3.5 × 10^−3^	3.4 × 10^−3^
88Sasada^52^	6421.07 − 6588.37	281/0/281/0	2.7 × 10^−3^	4.5 × 10^−3^
00LeRoHuMa^62^	11 396.04 − 13 018.40	513/0/513/0	7.0 × 10^−3^	7.8 × 10^−3^

Characteristics related to the observed spectra include the number of measured (*A*), floating (*F*), validated (*V*), and deleted (*D*) transitions, as well as *ΔU*^CSU^ and *ΔU*^MSU^, the average ‘claimed source’ and the average ‘MARVEL-suggested source’ expanded uncertainties of the transitions, respectively.

The usual IUPAC-recommended convention to label the states and the bands of the HCN molecule has been followed (see also Sec."Notation, quantum numbers, and selection rules"). The vibrational states and the related transitions are labeled as ${v}_{1}{v}_{2}^{{l}_{2}}{v}_{3}\equiv ({v}_{1}\,{v}_{2}\,{l}_{2}\,{v}_{3})$ and $(v^{\prime}_{1}\,v^{\prime}_{2}\,l^{\prime}_{2}\,v^{\prime}_{3})-(v^{\prime\prime}_1 \,v^{\prime\prime}_2 \,l^{\prime\prime}_{2}\,v^{\prime\prime}_3 )$, respectively. In the MARVEL input file, the rovibrational states have the label (*J* *v*_1_ *v*_2_ *l*_2_ *v*_3_ (*e*/*f*)) (see the Supplementary Information for this paper). In some older papers reporting transitions for H^12^C^14^N, the *v*_1_ and *v*_3_ vibrational quantum numbers were swapped, as the authors of 53DoSh^22^, 56AlTiPl^25^, 56DaTh^24^, 60RaSkEaWi^28^, 61RaEaRaWi^29^, 64MaBl^31^, 64MaPlTh^32^, 70MaOlSa^36^, 80GeGo^46^, 85KyChElDa^48^, and 88Sasada^52^ followed an earlier, non-IUPAC-supported convention to number the vibrational modes. To have a consistent input dataset in all these cases, we reversed the order of the two vibrational quantum numbers to maintain consistency with transitions reported in newer sources.

#### Comments on the literature sources utilized

60BrHoNiNa^27^: since the uncertainty of the measured line positions is not explicitly stated in this article, an expanded uncertainty of 0.01 cm^−1^ was assumed based on the data provided for all its transitions. Furthermore, data for the (0 3 1 0) − (0 1 1 0) band are reported twice, once with rotationless parity *c* and once with *d*. Comparing these transitions with data in the HITRAN2020 database^129^, it was found that *c* corresponds to rotationless parity *f* and *d* corresponds to *e*.

64MaBl^31^: typographical errors were identified in lines 64MaBl.102 and 64MaBl.200. The wavenumbers reported for these lines are 1919.711 and 3985.66 cm^−1^, respectively, whereas the correct values, in accordance with the HITRAN2020 database^129^, are 1999.711 and 3984.668 cm^−1^, respectively. For transitions of Table II of ref. ^31^ associated with the Q branch of the (0 3 1 0) − (0 0 0 0) band, it was determined that all transitions are actually located around 2100 cm^−1^ and not around 2300 cm^−1^, as originally reported.

72YiRa^39^: the transition labeled as 72YiRa.44 contains a typographical error. The correct wavenumber value is 664.6732 cm^−1^ instead of 663.6732 cm^−1^.

86ChTiKu^49^: in the (1 0 0 0) − (0 0 0 0) band, the authors appear to have interchanged the wavenumbers of the P23 and R23 transitions. For the (1 2 2 0) − (0 2 2 0) band, the article provides two separate tables: one for transitions with *e* rotationless parity and another for transitions with *f* rotationless parity. However, comparison with the HITRAN2020 database^129^ revealed that the parity assignments were switched: the transitions labeled as *e* components actually correspond to *f* components, and vice versa.

87ChKwKu^50^: a comparison of these transitions with the HITRAN2020 database^129^ revealed that the rotationless parity of 35 transitions was swapped in the case of the (0 4 2 0) − (0 1 1 0) band, including 20 transitions from P3e to P23e and 15 transitions from R2e to R17e.

89DuGa^53^: the transition labeled 89DuGa.192 contains a typographical error. It was originally assigned to a band between 697 and 699 cm^−1^, but its reported wavenumber is 705.72443 cm^−1^, which falls outside this range. A comparison with the HITRAN2020 database^129^ confirmed that the correct value for this transition is 697.15712 cm^−1^; thus, we changed the wavenumber of this transition in the input file to MARVEL.

00MaMeKlWi^61^: the expanded uncertainty of most of the transitions reported in this source is less than 0.001 cm^−1^.

03ZeAmAhBr^67^: There is a typographical error in the transition labeled 03ZeAmAhBr.154. The correct frequency value of this line is 1,871,448.619 MHz instead of the reported 187 148.619 MHz.

08MeWiWi^70^: The expanded uncertainty of most of the transitions is 0.002 cm^−1^; however, in approximately 1400 cases, the observed – calculated values are larger than 0.005 cm^−1^. In these cases, instead of the published uncertainties, we used the observed – calculated values as initial uncertainties.

#### Comments on literature sources not utilized

53DoSh^22^: this source includes 746 transitions within the spectral range of 5396 — 18,297 cm^−1^, spanning 17 vibrational bands. Due to the relatively large expanded uncertainty of 0.02 cm^−1^, all transitions from this source were excluded from the final analysis. Instead of 53DoSh, a more reliable source, 00MaMeKlWi^61^, containing the same transitions, was used.

56DaTh^24^: this source contains 160 transitions within the spectral range of 2052 − 2879 cm^−1^, spanning two vibrational bands. The article does not specify any uncertainty values. Based on the data and the number of significant digits in the reported wavenumbers, an expanded uncertainty of 0.05 cm^−1^ was assumed. Due to this relatively large uncertainty, all transitions from this source had to be excluded from the final analysis. In the end, only the significantly more accurate source, 00MaMeKlWi^61^, containing the same transitions, was used during the MARVEL analyses.

74BeEd^41^: inclusion of the pure rotational and rovibrational Raman transitions of this source in the overall dataset resulted in a significant increase in the number of conflicts among the measured transitions; therefore, all transitions from this source were excluded from the final MARVEL analysis.

56AlTiPl^25^: this source includes 176 transitions within the spectral range of 2753 − 6597 cm^−1^, spanning four vibrational bands. The article does not specify measurement uncertainties; thus, based on the provided data, an expanded uncertainty of 0.01 cm^−1^ was assumed, which is relatively large. Additionally, the same transitions were measured with higher accuracy in a more recent study, 88Sasada^52^. As a result, all 56AlTiPl transitions were excluded from the final dataset.

64MaPlTh^32^: this source includes 104 transitions in the range of 3171 − 3340 cm^−1^, across two vibrational bands. However, 21 transitions were reported without specifying their parity, causing these lines to be counted twice in the dataset, once with *e* rotationless parity and then with *f*, bringing the total to 124 transitions. The reported expanded uncertainty for this source is 0.15 cm^−1^, which is relatively high. Since more accurate studies, including 00MaMeKlWi^61^, 86ChTiKu^49^, and 00PiGu^59^, have analyzed the same bands, all transitions from this source were excluded from the final dataset.

02MoMaWiWi^144^: this article primarily focuses on DCN, but the authors note that their Supplementary Material includes transitions for HCN. Adding these transitions to the input dataset caused significant distortions. Upon further investigation, it was discovered that the transitions were actually for DCN and not for HCN.

#### Comments on references 61 and 70

The two largest and most important sources of experimental H^12^C^14^N transitions included in the MARVEL analysis of this study are due to Mellau and co-workers^61,70^. To appreciate the present study, it is worth commenting on these sources in a somewhat detailed way.

In both papers^61,70^, the authors used carefully designed effective Hamiltonians to obtain molecular eigenenergies. The approach chosen is particularly important when dealing with emission spectra, where each spectral feature is a composite of several transitions. The analysis performed in refs. ^61^ and ^70^ was designed to help solve this resolution issue. Thus, according to the main author, the calculated eigenenergies are supposedly an order of magnitude more accurate than the transition frequencies of the peak list generated from the measured spectra. In the MARVEL approach, one cannot expect to make such a correction; the empirical rovibrational energies corresponding to less accurately measured transitions may appear as outliers and have large uncertainties. Nevertheless, MARVEL should help to check the true accuracy of the eigenenergies coming from effective Hamiltonians.

When feasible, spectroscopists track each band to the last detectable peak down to the noise level. The *J* value of the last “observable” peak, call it ${J}_{\max }$, provides important information about the reliability of the data. ${J}_{\max }$ means that the last few peaks before it are not as accurate as the rest of the data, and that the next few transitions above ${J}_{\max }$ can still be predicted with acceptable accuracy. The idea behind this is that some less accurate data is better than none at all when effective Hamiltonians are employed. This is again not something one can use in a MARVEL approach. If, in a new publication, the data with a higher ${J}_{\max }$ is remeasured, the improved spectroscopic parameters provide improved data for these states, as well. Nevertheless, the data reported in these two publications were used successfully in our MARVEL analysis, but transitions were only considered up to the ${J}_{\max }$ value given by the authors. We decided not to use any of the transition values reported where the rotational quantum number is beyond ${J}_{\max }$.

The Supplementary Material of ref. ^70^ contains (a) screenshots, from which something like a peak list can be extracted, and (b) tables, containing predicted transitions and placeholders for transitions where it was not possible to extract precise information from the spectrum. Spectral features, where some of the components were already known, helped to reduce the degrees of freedom of the fitting, allowing more information to be read from the spectra, are denoted there by the letter ‘a’ (for ‘adjusted’). We have included these data in the MARVEL analysis, despite the fact that the resulting energies are less accurate than the data presented in the paper and may not represent the final result of ref. ^70^. Transitions with the descriptor ‘n’ in these tables were not included in our MARVEL input file.

#### Final comments

The number of observed/unique rovibrational transitions for H^12^C^14^N is 23 225/14 728. In line with previous MARVEL-based studies of measured and assigned rovibrational spectra, only about 2/3 of all the measured transitions are unique.

It is also important to point out the relatively large number of measured transitions that had to be deleted during the MARVEL analysis. The largest number of deleted transitions can be found in the following sources: 96 in 00PiGu^59^, 33 in 72YiRa^39^, 42 in 00MaMeKlWi^61^, and 13 in 60BrHoNiNa^27^.

It is perhaps even more important to note the relatively large number of transitions forming floating components, especially orphans, an apparent result of deleting the transitions with a label ‘n’ in the case of ref. ^70^. Transitions in floating components cannot be validated based on the information available from a MARVEL analysis.

## Supplementary information

Description of Additional Supplementary Files

Supplementary Data 1

Supplementary Data 2

Supplementary Data 3

Supplementary Data 4

## Data Availability

The MARVEL input files (Supplementary Data 1 and Supplementary Data 2), including all the transitions, and the MARVEL output file (Supplementary Data 3), with all the empirical energy levels, are supplied as Supplementary Information to this paper. The file Supplementary Data 4 explains the contents of these files. Both the measured transitions and the empirical energy levels have expanded (two-sigma) uncertainties attached to them.
