# Supplementary material for: Measured active rotational-vibrational energy levels (MARVEL) analysis of high-resolution rovibrational spectra of H12C14N
Source: Commun Chem. 2026 Apr 28;9:231. doi: 10.1038/s42004-026-02031-5 (PMC13332032; doi:10.1038/s42004-026-02031-5)
Supplement: Supplementary file 1 — Description of Additional Supplementary Files [file 42004_2026_2031_MOESM1_ESM.pdf]

Description of Additional Supplementary Files:

File name: Supplementary Data 1

Description: Contains all the transitions data, utilized during the MARVEL analysis, and has the following format:

transition wavenumber (in cm-1, deleted lines have negative wavenumber entry),

initial uncertainty (in cm-1),

final MARVEL uncertainty (in cm-1),

quantum numbers (upper state, lower state, see the EnergyLevels files),

tag.linenumber.

File name: Supplementary Data 2

Description: Contains the tag corresponding to the given data source unit of the transition entry.

File name: Supplementary Data 3

Description: Contains the empirical (MARVEL) energy levels in the following format:

quantum numbers (J v1 v2 l2 v3 p),

energy level (in cm-1),

uncertainty (in cm-1),

nbTr: number of transitions which determine the given empirical rovibrational energy.

File name: Supplementary Data 4

Description: Supplementary Information for the paper Measured Active Rotational-Vibrational Energy Levels (MARVEL) Analysis of High-Resolution Rovibrational Spectra of H12C14N

by Waed O. H. Al-Nashash, Ala'a A. A. Azzam, Sana A. E. Abzakh, Dunia Alatoom,

Mohammad Taha I. Ibrahim, Jonathan Tennyson, Tibor Furtenbacher, and Attila G. Cs\'asz\'ar
